# Characterization of single disseminated prostate cancer cells reveals tumor cell heterogeneity and identifies dormancy associated pathways

**DOI:** 10.18632/oncotarget.2480

**Published:** 2014-09-16

**Authors:** Lisly Chéry, Hung-Ming Lam, Ilsa Coleman, Bryce Lakely, Roger Coleman, Sandy Larson, Julio A. Aguirre-Ghiso, Jing Xia, Roman Gulati, Peter S. Nelson, Bruce Montgomery, Paul Lange, Linda A. Snyder, Robert L. Vessella, Colm Morrissey

**Affiliations:** ^1^ Department of Urology, University of Washington, Seattle, WA; ^2^ Divison of Human Biology, Fred Hutchinson Cancer Research Center, Seattle, WA; ^3^ Division of Hematology and Oncology, Department of Medicine and Department of Otolaryngology, Tisch Cancer Institute, Black Family Stem Cell Institute, Ichan School of Medicine at Mount Sinai, New York, NY; ^4^ Divison of Public Health Sciences, Fred Hutchinson Cancer Research Center, Seattle, WA; ^5^ Department of Medicine, University of Washington, Seattle, WA; ^6^ Department of Veterans Affairs Medical Center, Seattle, WA; ^7^ Janssen Research and Development, LLC, Spring House, PA

**Keywords:** Prostate cancer, dormancy, metastasis, p38, gene expression

## Abstract

Cancer dormancy refers to the prolonged clinical disease-free time between removal of the primary tumor and recurrence, which is common in prostate cancer (PCa), breast cancer, esophageal cancer, and other cancers. PCa disseminated tumor cells (DTC) are detected in both patients with no evidence of disease (NED) and advanced disease (ADV). However, the molecular and cellular nature of DTC is unknown. We performed a first-in-field study of single DTC transcriptomic analyses in cancer patients to identify a molecular signature associated with cancer dormancy. We profiled eighty-five individual EpCAM^+^/CD45^−^ cells from the bone marrow of PCa patients with NED or ADV. We analyzed 44 DTC with high prostate-epithelial signatures, and eliminated 41 cells with high erythroid signatures and low prostate epithelial signatures. DTC were clustered into 3 groups: NED, ADV_1, and ADV_2, in which the ADV_1 group presented a distinct gene expression pattern associated with the p38 stress activated kinase pathway. Additionally, DTC from the NED group were enriched for a tumor dormancy signature associated with head and neck squamous carcinoma and breast cancer. This study provides the first clinical evidence of the p38 pathway as a potential biomarker for early recurrence and an attractive target for therapeutic intervention.

## INTRODUCTION

An estimated 29,480 men will die from PCa in the United States in 2014 [1]. The majority of patients who present with clinically localized prostate cancer (PCa) proceed to primary treatment with curative intent [2]. Patients diagnosed with organ-confined disease have decreased recurrence and increased overall survival when compared to patients with cancer that has metastasized [3, 4]. Disseminated disease has a poor prognosis as no curative treatments exist.

Not all cells that escape from the primary tumor form clinical metastases. Primary tumor cells that enter the vasculature are called circulating tumor cells (CTC). The majority of CTC are unable to establish in peripheral tissues and will die in the vasculature [5-8]. When CTC are able to exit the vasculature and establish residency in distal tissues such as the lung, liver, and bone marrow (BM), they are termed disseminated tumor cells (DTC). Several solid tumors, including PCa, have been postulated to have DTC that can remain in a state of dormancy for an extended period of time [9-11]. In PCa, these dormant DTC can become active years after treatment of the primary tumor as exhibited by late PSA recurrences [12].

Dormant DTC pose a difficult clinical problem. First, they are difficult to detect and they may be present in patients that are asymptomatic or in those with active disease. Second, in both patient groups, dormant DTC are likely resistant to most treatments, which target cells undergoing replication and/or have identifiable and targetable signaling pathways. Third, dormant DTC do not express any known biomarker that is detectable via non-invasive or minimally invasive techniques such as urine collection or peripheral blood draw. Fourth, there are not any FDA approved tests that can readily identify and isolate dormant DTC.

The characterization of dormant DTC and identification of the pathways involved in tumor cell dormancy are needed to determine which patients are likely to have dormant DTC that have the potential to eventually progress to clinical metastases, and to identify potential targets for therapeutic intervention. In this study, our objectives were to identify DTC in patients with PCa, characterize DTC at the single-cell level, analyze the heterogeneity of DTC within and among patients, and identify markers of tumor cell dormancy.

## RESULTS

### Sample analysis

BM was drawn from five patients diagnosed with PCa and no evidence of disease following primary treatment (NED) and six patients with metastatic disease or biochemical recurrence following primary treatment (ADV). The characteristics of the patients in each group are displayed in Supplemental Table 1. One hundred and five individual EpCAM^+^/CD45^−^ cells were isolated from the BM aspirates of the 11 PCa patients. Briefly, BM aspirates were placed over Ficoll-Isopaque, the mononuclear cell layer containing DTC underwent immunomagnetic negative selection to eliminate leukocytes, megakaryocytes, and platelets and then positive selection for epithelial cells. The enriched population was subjected to immunostaining and EpCAM^+^/CD45^−^ cells were isolated using a micromanipulator. Quality control for the genetic material isolated and amplified from isolated single cell DTC was performed as previously detailed [13]. Twenty cells were removed due to more than 25 percent of probes having missing values or to poor microarray hybridization signals, ending up with 85 cells for gene expression analyses [Supplemental Figure 1]. Thirty-nine cells were analyzed from NED patients and 46 cells were analyzed from ADV patients. Each cell that was isolated was given an EpCAM intensity [Supplemental Figure 2].

**Figure 1:**
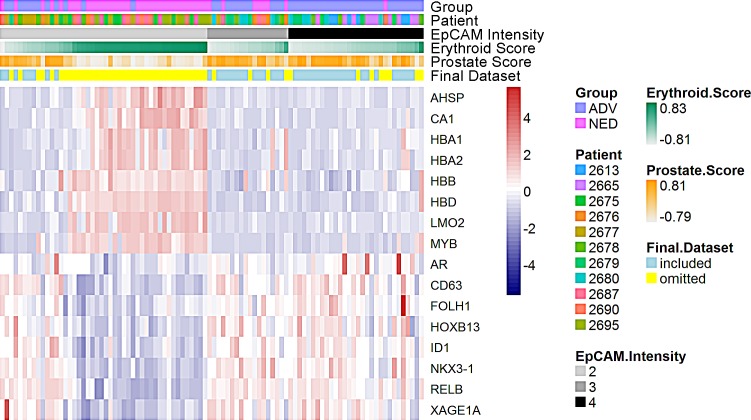
Identification of an erythroid progenitor-like signature and a prostate specific signature The gene expression signature of single cells isolated from BM aspirates of patients with no evidence of disease [NED, pink along the top bar, 39 cells; 4 patients] and advanced patients [ADV, purple along the top bar, 46 cells; 6 patients] is shown in each column. Each color in the second horizontal bar represents a different patient. Cells are grouped according to EpCAM score indicated in the third horizontal bar. AHSP, CA1, HBA1, HBA2, HBB, HBD, LMO2, and MYB were used to identify an erythroid progenitor-like signature. The fourth horizontal bar denotes the erythroid progenitor-like score for each cell. AR, CD63, FOLH1, HOXB13, ID1, NKX3-1, RELB, and XAGE1A were used to identify a prostate specific signature. The fifth horizontal bar denotes the prostate specific signature intensity for each cell. Cells used or excluded from further analysis are depicted in blue or yellow, respectively, in the sixth horizontal bar.

### Identification of an erythroid progenitor-like signature

Upon examination of the gene expression array data of the 85 individual cells, there was a subset of cells with high expression of genes that are associated with erythroid progenitor-like cells [AHSP, CA1, HBA1, HBA2, HBB, HBD, LMO2, and MYB] [Figure 1]. Immature erythroid progenitor cells in the BM have been previously documented to transiently express EpCAM and E-cadherin [14, 15]. To ensure the cells used for further analysis were prostate epithelial cells, we defined a dual gene expression signature for selection: EpCAM^+^/CD45^−^ cells were defined as prostate epithelial cells if there was (i) low expression of erythroid progenitor-like signature including AHSP, CA1, HBA1, HBA2, HBB, HBD, LMO2, and MYB and (ii) high expression of prostate epithelial genes including AR, CD63, FOLH1, HOXB13, ID1, NKX3.1, RELB, and XAGE1A. NKX3.1 was the most consistent marker differentiating EpCAM+ prostate cells from EpCAM+ cells with the erythroid progenitor-like signature [Figure 1]. All of the cells fitting the two criteria of prostate epithelial cells had elevated expression of NKX3.1. Of the 85 cells originally isolated, 41 were excluded due to a high erythroid progenitor-like score (34/41 DTC) or a low prostate epithelial score including NKX3.1 (7/41 DTC) [Figure 1; Supplemental Figure 1]. Among the 34 cells that present a high erythroid progenitor-like score, 32 cells had an EpCAM intensity of 2^+^ and were CD45^−^. Only 2 cells that were CD45^−^ with an EpCAM intensity of 4^+^ had a high erythroid progenitor-like score. The remaining 44 cells, 7 cells from 4 patients in the NED group and 37 cells from 6 patients in the ADV group were considered PCa DTC and further analyzed [Supplemental Figure 1].

### DTC display heterogeneity within and between NED and ADV patients

**Figure 2:**
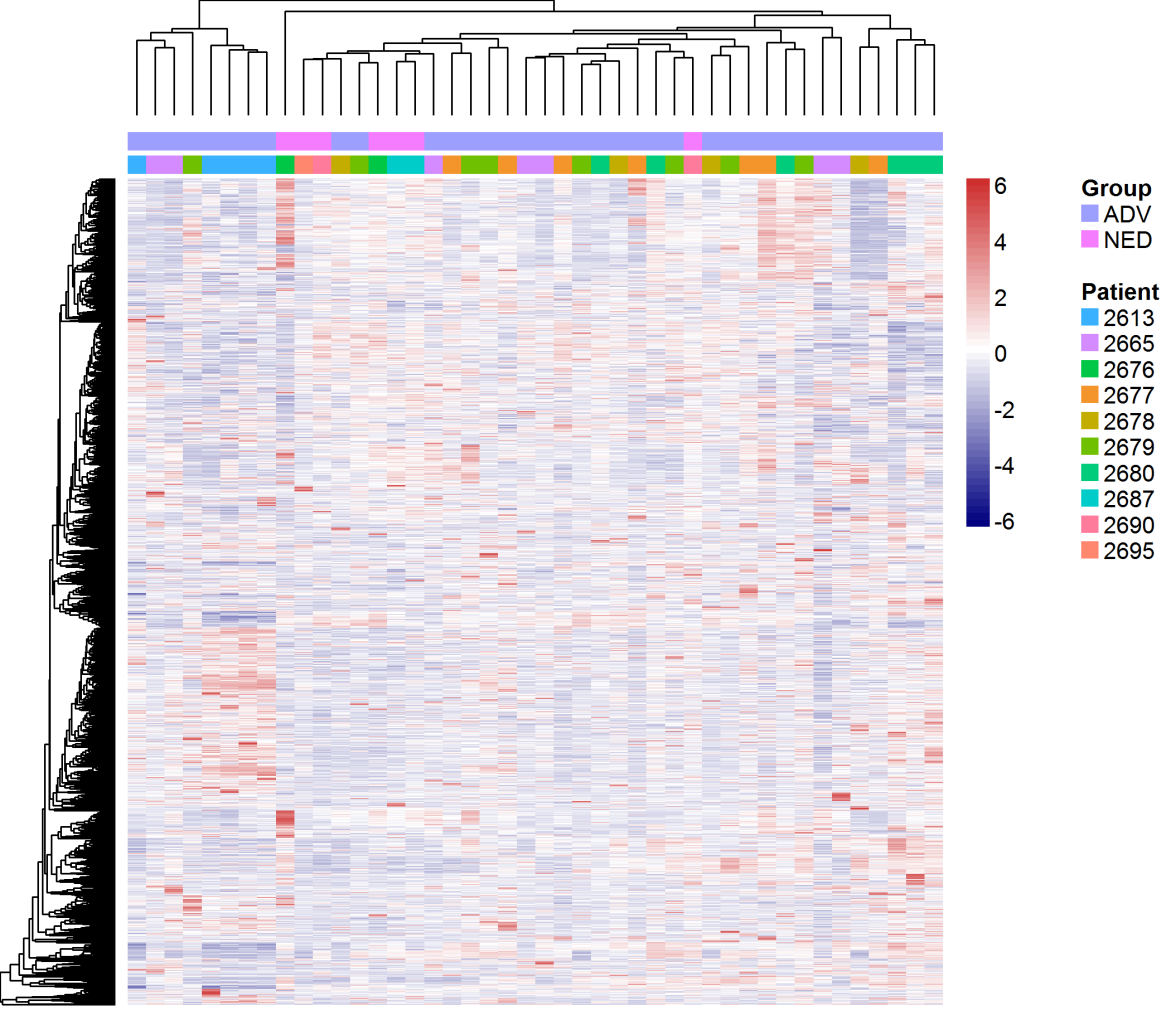
Cluster analysis of 5,000 most variable genes suggests heterogeneity between and within patient samples An unsupervised cluster analysis of the 5,000 most variable genes from individual prostate cancer DTC isolated from the BM of patients with no evidence of disease [NED] (pink in the top bar, 2676, 2687, 2690, 2695) and advanced disease [ADV] (purple in the top bar, 2613, 2665, 2677, 2678, 2679, 2680). Each color in the second bar represents a different patient.

Cluster analysis of the 5000 most variable genes between the NED and ADV groups exhibited heterogeneity within DTC taken from patients in both the NED and ADV groups [Figure 2]. Not all DTC taken from patients in the NED group clustered together. DTC from NED and ADV patients were dispersed throughout the cluster. Additionally, not all DTC from the same patient cluster together. Of the 6 ADV patients included in the analysis, patient 2613_ADV showed 4 out of 5 DTC clustered together, however the remaining 5 ADV patients had DTC dispersed throughout the cluster [Figure 2].

### Identification of two gene signatures in DTC from ADV patients

**Figure 3:**
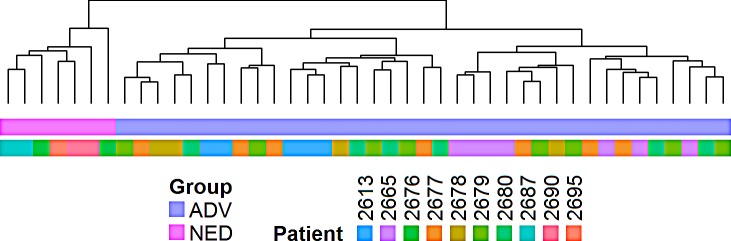
Cluster analysis of the the top 50 upregulated and 50 downregulated genes segregates individual prostate cancer DTC into 3 categories Hierarchical cluster analysis of individual prostate cancer DTC obtained from the BM based on the top 50 and bottom 50 genes differentially expressed between patients with no evidence of disease [NED] and patients with advanced disease [ADV]. Cells on the right of the figure were obtained from ADV patients (purple on the top horizontal bar) clustered into 2 groups. Cells on the left of the figure were obtained from NED patients (pink on the top horizontal bar) clustered into a single group. Each color in the second bar represents a different patient.

**Figure 4:**
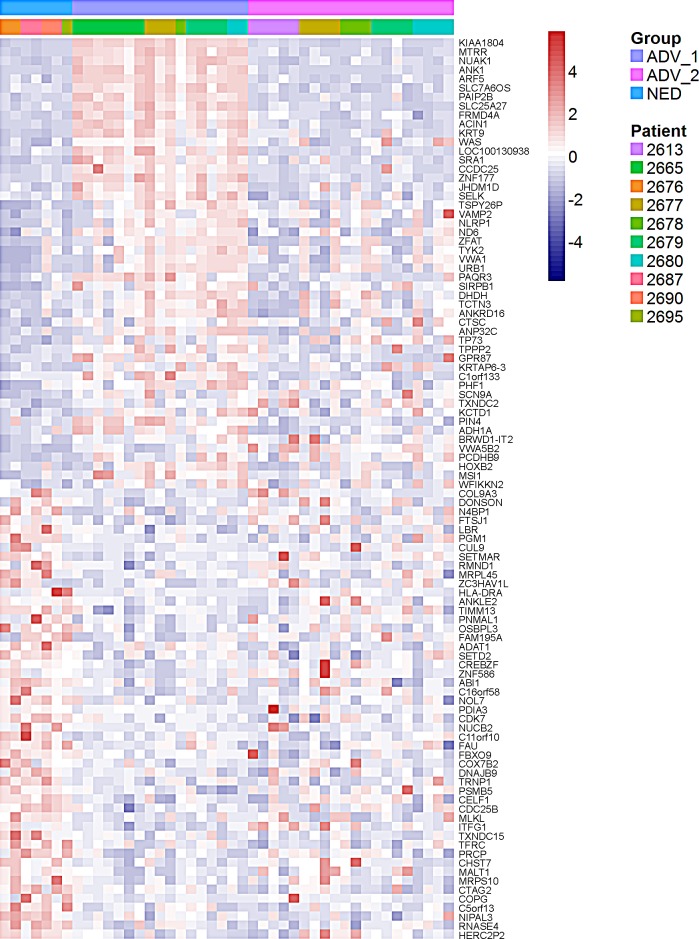
Heat map demonstrates individual prostate cancer DTC from patients with no evidence of disease [NED] are similar to a subset of prostate cancer DTC from advanced patients [ADV] A supervised analysis of the top 50 and bottom 50 genes differentially expressed between NED patients and ADV_1 patients. Cells on the right of the figure were obtained from ADV patients and clustered into 2 groups (ADV_1: purple on top horizontal bar and ADV_2: pink on top horizontal bar). Cells on the left of the figure were obtained from NED patients and clustered into a single group (blue on top horizontal bar). Each color in the second bar represents a different patient.

Clustering analyses based on the top 50 upregulated and 50 downregulated genes between the NED and ADV patient groups showed the DTC obtained from NED patients formed a single NED cluster [Figure 3]. DTC isolated from ADV patients formed two clusters (ADV_1 and ADV_2 clusters) in which ADV_1 had a gene expression profile that differed from that of the NED cluster, whereas ADV_2 had a gene expression profile that was similar to that of the NED cluster [Figure 4]. These results suggest that ADV patients harbor at least two distinct populations of DTC that may be associated with different ADV phenotypes (ADV_1 and ADV_2). Notably, all of the DTC isolated from patient 2613_ADV were in the ADV_2 cluster; all of the DTC isolated from patient 2665_ADV were in the ADV_1 cluster. The remaining 4 ADV patients had DTC in both the ADV_1 and ADV_2 clusters. Additionally, we determined that DTC EpCAM intensity levels do not distinguish between the ADV_1 and ADV_2 groups [Supplemental Figure 3].

### Components of the p38 pathway are differentially expressed in NED DTC and a subset of ADV DTC

**Figure 5:**
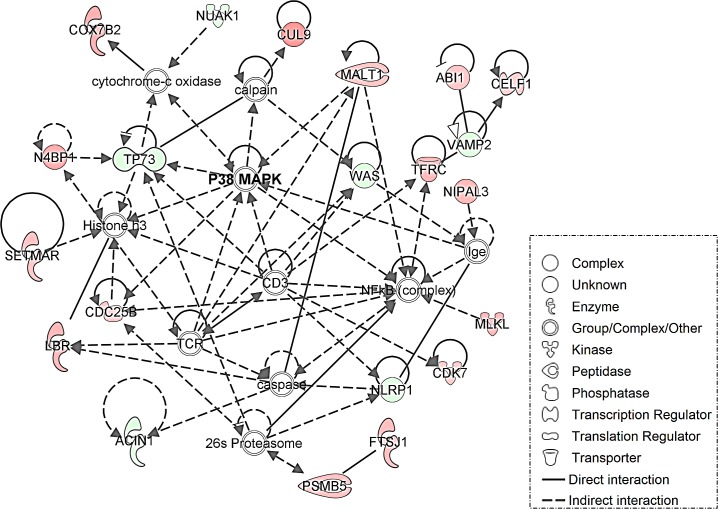
Genes associated with the p38 pathway are altered between no evidence of disease [NED] and a subset of advanced disease [ADV_1] prostate cancer DTC Ingenuity Pathway Analysis identified the p38 stress response pathway as the top biological pathway altered in NED prostate cancer DTC vs. a subset of the advanced prostate cancer DTC [ADV_1]. Red indicates an increase in gene expression, green a decrease. Genes in uncolored nodes were not identified as differentially expressed in our microarray analyzes but were relevant to and therefore incorporated into individual networks based on the IPA database.

Gene set enrichment analysis identified pathways differentially regulated between the NED and ADV_1 clusters. In the ADV_1 cluster, genes involved in cytokine and chemokine signaling pathways were upregulated [p<0.001], whereas genes involved in the epithelial to mesenchymal transition and kinase inhibitor activity pathways were downregulated [p=0.013] and [p=0.007] respectively. Ingenuity Pathway Analysis (IPA) conducted on the 50 most upregulated genes and the 50 most downregulated genes showed that the p38 pathway was the top differentiating pathway involved between the NED and ADV_1 clusters [Figure 5]. We conducted a secondary analysis in an attempt to identify additional pathways altered between NED and ADV_1. We excluded the p38 pathway by looking at pathways that ranked second and third, both pathways involved ubiquitin C (UBC)-associated proteins. Furthermore, we used an alternate method by eliminating the twenty p38-associated genes and re-ran the IPA analysis. Again, UBC-related pathways showed up as the top two most significant pathways [Supplemental Figures 4 and 5]. Based on these two analyses, the results suggest the p38 pathway is the dominant pathway that represents the top 50 and bottom 50 most differentially expressed genes. When we compared the NED vs. ADV and ADV_2 clusters, the top networks identified involved fibronectin, ERK1/2, and p38 [Supplemental Figure 6 and 7]. Activation of the p38 pathway has been associated with DTC dormancy for solid tumors including HNSCC [16], breast [17], and prostate [18]. Key genes, including KIAA1804 (MLK4β), MTRR, and NUAK1 are downregulated in the NED cluster when compared with the ADV_1 cluster [Figure 4]. All three of these genes are involved in regulation of the p38 pathway [19-21]. It is important to note that not all p38-associated genes identified in the gene signature were present in the IPA analysis tool for IPA analysis. PIN4, a gene implicated in cell cycle re-entry, was also downregulated in the NED cluster [22]. MALT1 and CDC25B, a positive regulator and an effector of the p38 pathway respectively [23, 24], were upregulated in the NED cluster when compared with the ADV_1 cluster (Figure 4). Collectively, these data showed a distinct gene expression pattern associated with a potential dormancy-inducing p38 stress activated kinase pathway.

### DTC from patients with no evidence of clinical recurrence display a distinct dormancy signature

**Figure 6:**
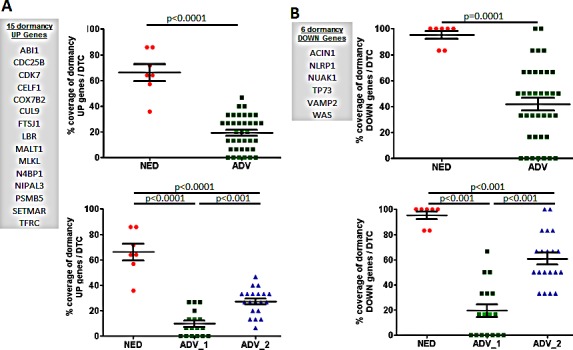
DTC analyses of p38 dormancy-associated genes A. DTC from patients with no evidence of disease [NED] were significantly enriched when compared to DTC from advanced patients [ADV] with 14 p38-associated genes that were upregulated in the top 50 differentially expressed genes in NED vs. ADV, and two subsets of advanced patient [ADV_1, and ADV_2] DTC. B. The 6 genes that were downregulated in NED DTC were significantly repressed vs. ADV, ADV_1, and ADV_2 DTC. Mean±SD is shown. NED was compared to ADV, ADV_1, or ADV_2, and p<0.05 was considered statistically significant.

**Figure 7:**
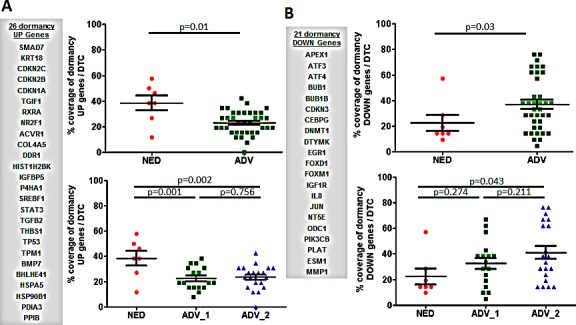
DTC analyses of dormancy-associated genes A. A dormancy-related signature identified through previous dormancy studies (12-15) was used to compare to the signature obtained from the gene expression arrays. A. DTC from patients with no evidence of disease [NED] were significantly enriched for 26 dormancy-related genes that are upregulated in NED vs. DTC in advanced patients [ADV], and two subsets of advanced patient [ADV_1, and ADV_2] DTC. B. Twenty-one genes that were downregulated in a dormancy-related signature were not repressed in NED vs. ADV or when compared to ADV_1 or ADV_2 DTC. Mean±SD is shown. NED was compared to ADV, ADV_1, or ADV_2, and p<0.05 was considered statistically significant.

The p38 stress response pathway activated by TGFβ2 and other cues has been proposed as a regulator of tumor cell dormancy [10, 16, 18, 25, 26]. DTC from patients with NED were significantly enriched for p38-associated genes when compared to DTC from ADV patients. Our bioinformatics IPA analysis identified p38 stress response genes as being differentially regulated between the NED and ADV_1 DTC. Focusing on the top 50 upregulated and 50 downregulated genes, 15 p38-associated genes were upregulated in the top 50 upregulated genes and 6 p38-associated genes were significantly repressed in the top 50 downregulated genes in NED vs. ADV DTC. The 15 upregulated genes were significantly enriched in NED vs. ADV or the ADV_1 and ADV_2 groups (NED vs. ADV, p<0.0001; NED vs. ADV_1, p<0.0001; and NED vs. ADV_2, p<0.0001) [Figure 6A]. The six downregulated genes were significantly enriched in NED vs. ADV or the ADV_1 and ADV_2 groups (NED vs. ADV, p=0.0001; NED vs. ADV_1, p<0.001; and NED vs. ADV_2, p<0.001) [Figure 6B]. Interestingly, DTC in ADV_2 showed a significant enrichment of the dormancy upregulated and downregulated genes than the DTC in ADV_1 [Figure 6A and B]. We conclude that this analysis identified 21 genes (up- and downregulated) that were associated with p38 signaling and indolent behavior in NED DTC. We next tested whether a previously identified p38 dormancy signature was differentially enriched in NED and ADV DTC. We found that DTC from NED patients were significantly enriched with the 26 genes that were upregulated in a previously identified dormancy-associated signature (NED vs. ADV, p=0.01; NED vs. ADV_1, p=0.001; NED vs. ADV_2, p=0.002) [Figure 7A]. However, the p38-regulated signature did not distinguish between ADV_1 and ADV_2 groups [Figure 7A]. The 21 genes that were downregulated in the published dormancy-associated signature showed no significant difference between the NED vs. ADV_1, however statistical significance was reached when compared between NED vs ADV_2 (p=0.43) and NED vs ADV as a whole [p=0.03, Figure 7B]. Using two different approaches our data reveals that DTC from NED patients carry a p38-associated signature consistent with its role in induction of prolonged quiescence and DTC dormancy in experimental systems.

## DISCUSSION

We identified and characterized a definitive set of DTC in patients with a high prostate epithelial score and a low erythroid progenitor-like score. The majority of the PCa DTC had an EpCAM score of 3+ or 4+, with only a few cells displaying an EpCAM score of 2+. We profiled individual cells instead of pools of cells to avoid the inclusion of any contaminating cell types in our signatures, which would confound data analyses for definitive PCa DTC. Until further evidence is available to suggest that the predominantly EpCAM 2+ cells with a low prostate epithelial score and a high erythroid-progenitor-like score are associated with PCa, we determined that based on the extensive cellular plasticity present in these cells that they should be removed from our analysis. Of note, it is unlikely that these erythroid progenitor-like cells are present in the blood contaminating the analysis of CTC as erythropoiesis usually occurs in the BM, however the possibility should not be ruled out, e.g. extrameduallary erythropoiesis can occur in myelofibrosis [27].

DTC from most patients (except one patient) unsurprisingly were heterogeneous between and within patient samples at the transcriptional level, regardless of disease type (i.e. NED or ADV) [28, 29]. One could speculate that DTC obtained from one patient may all arise from a single micrometastasis (e.g. patient 2613), providing some clonality and a similar microenvironment for each of these cells. However, in the other patients, cells may have been isolated from different micrometastases or individual cells present in the BM and this would explain why some but not all cells from patients cluster together. Similar reasoning/hypothesis may explain why ADV_2 clusters with NED, with ADV_2 and NED cells being present as individual cells and ADV_1 cells being isolated from groups of cells. Of note, when we clustered individual DTC based on the expression of the top and bottom 50 differentially expressed genes in NED and ADV_1, we observed a greater degree of patient-associated clustering of the individual DTC [Figure 4]. This suggests the level of heterogeneity observed will depend on the number of genes, or more specifically the biological process assessed in the signature.

DTC in ADV patients could be clustered into two groups: ADV_1 and ADV_2. Of note, we observed no difference between biochemical and radiographic disease states in ADV patient samples when compared to NED patient samples (data not shown). Among the patients in the ADV category, 4 of 6 had DTC in both the ADV_1 and ADV_2 clusters. This indicates that the DTC in the patients with clinically advanced disease harbor cells in different biological states. For the top 50 and bottom 50 genes differentially expressed between NED and ADV_1 categories, surprisingly the ADV_2 DTC were similar to the NED DTC. This does not necessarily mean the ADV_2 DTC were also in a state of dormancy, but the similarity in the gene expression pattern suggests potential concordance in their behavior. This is of clinical importance, as the current therapies for treatment of advanced PCa targets cells that are actively replicating. Cells in a state of dormancy/quiescence would not be affected by these treatment strategies, and they could potentially exit dormancy and become active metastases after treatment has concluded. If the ADV_2 DTC possess some of the hallmarks of the DTC from the NED patients, it could be hypothesized that a subpopulation of PCa cells in the BM of the ADV patients are in a dormant/quiescent state. Further, the challenge for cure in these patients would rely on treating both the active and dormant PCa DTC.

The identification of clinically-relevant pathways involved in PCa dormancy in patients is needed to develop dormancy models, identify robust markers and design effective treatment strategies. The activation of the p38 pathway has been identified to promote and maintain tumor dormancy [10, 16, 18, 25, 26]. A recent report demonstrated that PCa stem-like cells in the BM enter a p38-dependent dormancy state in an animal model, however the clinical relevance of the p38 pathway in PCa dormancy is yet to be established [18]. In our study, genes associated with the p38 stress response pathway were shown to be altered when we compared DTC from NED patients to a group of DTC from ADV patients. In addition to the genes identified in the IPA analysis, both KIAA1804 (MLK4β) and MTRR were downregulated in the NED DTC [Supplemental Table 2]. The mitogen-activated protein kinase family and specifically MLK4β has been shown to inhibit the p38 pathway [19]. MTRR is an electron transferase involved in methionine synthesis and has also been shown to inhibit the p38 pathway [21]. Thus, loss of these genes may be more permissive for persistent p38 activation in DTC in PCa patients.

NUAK1 is a protein kinase that has been linked to the activation of the p38 pathway and cell proliferation [20]. NUAK1 was found to be upregulated in the ADV_1 DTC population. PIN4 was also upregulated in the ADV_1 DTC group compared to the NED DTC. PIN4 function is linked to cell proliferation [30]. MALT1, which showed upregulation in the NED DTC, is a cysteine protease whose function has been linked to activation of the p38 pathway [23]. MALT1 regulates p38 and CDC25B is a downstream target of p38 [24]. CDC25B, a phosphatase that activates the cyclin-dependent kinase CDC2, was upregulated in NED DTC. p38 phosphorylates CDC25B, which results in an inhibition of entry into the M phase of the cell cycle [24]. Elevated mRNA levels for CDC25B might be a compensatory mechanism for p38-induced growth arrest. While extrapolating from gene expression to function is not completely reliable, the elevated expression of these 6 genes discussed above suggests a role for the p38 stress-response pathway in the DTC isolated from the BM of the NED patients.

We developed a p38-associated dormancy signature based on the top 50 and bottom 50 differentially expressed genes between NED DTC and ADV_1 DTC. This signature, while biased, could clearly differentiate between NED DTC and ADV DTC. To further validate our findings we interrogated the DTC expression profiles for a dormancy signature previously developed from a HNSCC model [16, 25, 31] and a PCa model [18]. Dormancy upregulated genes were enriched in the NED DTC, suggesting that the dormancy signature could consistently identify dormant PCa cells present in the patient BM. Among the dormancy-up genes in NED DTC, TGFβ2 and BMP7 have been implicated specifically in dormancy of DTC in the BM [16, 18]. However, the dormancy downregulated genes could not distinguish between the NED, ADV_1 and ADV_2 DTC. One possible explanation for this is that solitary PCa DTC may be slow cycling or non-proliferative in nature, therefore the dormancy-down genes that are linked to proliferation may not be changing sufficiently. Furthermore, dormancy initiation and maintenance may require an induction of the p38 stress response pathway, which results in the activation of different downstream cell cycle effectors among different tumor types.

The lack of clinically-relevant models in the dormancy field is a challenge that needs to be overcome. New model systems involving TGF-beta and the perivascular niche are emerging and may be relevant for PCa DTC study if proven clinically relevant [16, 32]. Despite the clinical relevance, our study is restricted in its interpretation due to the limited number of EpCAM+ cells that can be isolated from NED patient BM and the fact that we were unable to further interrogate individual cells at the protein level or functionally to validate our findings. Nonetheless, our study provides the first clinical evidence supporting a role for the p38 pathway in dormancy in PCa DTC in patients, which supports further functional validation *in vitro* and *in vivo*.

In summary, this study illustrates a first-in-field transcriptomic signature from individual DTC in cancer patients. We established a gene signature to definitively identify PCa DTC from other EpCAM^+^/CD45^−^ cells that do not demonstrate typical prostate epithelial markers in the BM. We further demonstrated that patients with advanced disease may harbor at least two populations of DTC, in which one group of DTC is similar to those isolated from patients with NED for up to 18 years. We also validated our signature using a mechanistically modeled dormancy-associated signature from head and neck cancer supporting the hypothesis that the p38 pathway is associated with the dormant nature of these DTC in NED patients. Ultimately, in the clinical setting, the intent is to maintain DTC in a dormant state or to better understand dormancy such that active disseminated cells can be shifted to a state of dormancy, and hence delay the onset of symptomatic and lethal metastatic disease. Our results suggest that the p38 pathway may serve as a potential biomarker to monitor early recurrence and identifies the p38 axis as an attractive target for therapeutic intervention to regulate dormancy in PCa patients.

## METHODS

### Selection criteria for patients

Patients from 2 clinical categories were selected. Patients who had a diagnosis of adenocarcinoma of the prostate, had undergone a radical prostatectomy (RP), had not received neoadjuvant or adjuvant treatment and had a PSA that was undetectable (less than 0.1 ng/mL) for a minimum of 7 years (range 7-18 years) after RP, were considered to have no evidence of disease (NED). Patients with advanced disease (ADV) were defined as either having radiographic evidence of metastatic disease at the time of diagnosis of adenocarcinoma of the prostate or having biochemical recurrence (defined as two consecutive rises in PSA > 0.2 ng/mL in a patient who previously had a RP and an undetectable PSA), after primary curative therapies for adenocarcinoma of the prostate.

### Enrichment of DTC from the BM of PCa patients

All materials were acquired and used conforming to IRB-approved protocols at the University of Washington. DTC were isolated from BM samples of PCa patients as previously reported [13]. Briefly, ten ml of BM was aspirated from the posterior iliac crest into a 30 ml syringe containing 10 ml of 6% sodium citrate under local anesthesia. Processing of samples commenced within 1–2 hours and was completed within 5 hours. BM aspirates were separated using 15 ml of Ficoll-Isopaque (1.077 g/ml) (Accurate Chemical, Westbury, NY). Centrifugation yielded a mononuclear cell layer containing DTC, which underwent immunomagnetic selection with the MACS system (Miltenyi Biotec, Auburn, CA). Anti-CD45 and anti-CD61 antibodies were used first for negative selection to eliminate leukocytes, megakaryocytes, and platelets. Positive selection was then performed with immunomagnetic beads coated with anti-human epithelial antigen (HEA) antibodies to target epithelial cells.

### Isolation of individual DTC

For identification and isolation of DTC, the enriched population was subjected to immunostaining with fluorescein isothiocyanate labeled anti-BerEP4/EpCAM antibodies (Dako, Carpinteria, CA), which bind a different epitope on HEA than the anti-HEA antibody used for positive selection. A phycoerythrin conjugated anti-CD45 antibody was also added for identification of leukocytes. EpCAM^+^/CD45^−^ cells were kept on ice, viewed under fluorescent light, and intact cells with a clearly defined EpCAM labelled membrane were isolated using a micromanipulator. Based on historic testing of this method using the trypan blue exclusion assay, DTC picked at this stage of isolation are viable. Individual cells were lysed with 2 μl of WT-Ovation™ One-Direct Amplification System (NuGEN) lysis buffer and stored at −80^o^C for gene expression profiling.

### Amplification of total RNA from individual DTC

Total RNA was amplified from each sample using the WT-Ovation™ One-Direct Amplification System (NuGEN) according to the manufacturer’s directions. The use of an aluminum cooling block on ice facilitated the handling of the reaction tubes. The amplified cDNA products passed the purity test of showing Abs_260_/Abs_280_ ratios between 1.9 to 2.0, and a subset was analyzed on an Agilent Bioanalyzer using the RNA 6000 Pico LabChip with the mRNA Pico program to assess size distribution as previously described [13]. Post-SPIA modification and post-amplification work was performed in a separate workspace.

### Labeling and hybridization of amplified material on Agilent chips

Amplified cDNA from each sample were labeled using the BioPrime® Total Genomic Labeling System (Invitrogen™). Hybridization probes were prepared by combining 6 mg of Alexa Fluor® 3 labeled sample and 1 mg Alexa Fluor® 5 labeled reference and denatured at 95°C and hybridized at 63°C on Agilent Human 4x44K microarrays and processed following the manufacturer’s specifications. Arrays were scanned on an Agilent DNA Microarray Scanner [13].

### DTC Selection

We analyzed DTC from 5/9 NED patients. The remaining 4 patients all had DTC, but had too few to move forward and analyze. These samples were not processed as our goal was to obtain approximately 10 cells/patient to analyze tumor cell heterogeneity, whereas these patients only had 1-4 cells. We analyzed DTC from 6/8 ADV sampled patients. The DTC isolated from one patient were of poor quality, the other patient had no DTC observed in the bone marrow sample.

### Gene expression analysis

Data were loess normalized within arrays and quantile normalized among arrays in R using the Limma Bioconductor package. Samples with poor microarray hybridization signals were removed. Data were filtered to remove probes with mean signal intensities below 300. The Statistical Analysis of Microarray (SAM) program (http://www-stat.stanford.edu/~tibs/SAM/) was used to analyze expression differences between groups. Unpaired, two-sample t-tests were calculated for 11202 unique genes passing filters and controlled for multiple testing by estimation of q-values using the false discovery rate (FDR) method. Microarray data are deposited in the Gene Expression Omnibus database under the accession number GSE48995. To determine whether transcriptomic differences observed in ADV versus NED groups were enriched for genes within canonical pathways and Gene Ontology gene sets, the t-test results were subjected to Gene Set Enrichment Analysis (GSEA) using preranked mode with permutation testing of the gene sets to adjust for multiple hypothesis testing, generating an FDR. Global unsupervised, hierarchical clustering analysis was performed with the top 5000 most variable genes among all samples as determined by inter-quartile range (IQR). Unsupervised, hierarchical clustering of the top and bottom 50 most differentially expressed between NED and ADV groups based on SAM score identified subgroups ADV_1 and ADV_2. Erythroid progenitor-like and prostate enrichment scores were calculated using the Gene Set Variation Analysis (GSVA) Bioconductor package in R [33].

### Ingenuity Pathway Analysis

The top and bottom 50 most differentially expressed genes (total 100 genes) between NED, ADV, ADV_1 and ADV_2 groups were imported into Ingenuity Pathway Analysis (IPA, Ingenuity Systems; https://www.ingenuity.com) to identify biological pathways involved in different groups of DTC. IPA is a repository of biological interactions and functional annotations to demonstrate relationships between proteins, genes, complexes, metabolites, and drugs. The IPA knowledge library includes information from NCBI databases (EntrezGene, RefSeq, OMIM disease associations), microRNA-mRNA target databases, GWAS (Genome Wide Analysis Study) databases, and Kyoto Encyclopedia of Genes and Genomes (KEGG).

### Analysis of DTC expression profiles for the presence of a p38α/β-regulated dormancy signature

For the p38α/β dormancy-associated signature identified in the IPA, the enrichment for dormancy upregulated or downregulated genes for each DTC was calculated. Specifically, the percent of dormancy upregulated genes that were also upregulated in the DTC was scored as the percent coverage of dormancy UP genes induced for each individual DTC. The same was applied to the downregulated genes in the above-mentioned signature. Cutoffs were the same as those used for all normalization analysis. In all cases, differences in means were estimated using a linear regression model and statistical significance was evaluated using t-tests of appropriate model coefficients. For the previously identified tumor dormancy-associated signature, expression profiles of the NED, ADV, ADV_1 or ADV_2 subgroups of DTC were parsed for the genes upregulated and downregulated in a previously published tumor dormancy gene signature of 19 upregulated and 21 downregulated genes linked to p38α/β activation in a head and neck squamous cell carcinoma (HNSCC) model [16, 25, 31] or to BMP7 signaling in a PCa model [18]. The latter signature only added BMP7. This signature was expanded to incorporate an additional 7 genes that have been linked to *in vivo* quiescence of dormant HNSCC cells (Aguirre-Ghiso et al., unpublished). The percent of upregulated genes in the signatures that were also upregulated in the DTC was scored as the percent coverage of dormancy UP genes induced for each individual DTC. The same was applied to the downregulated genes in the above-mentioned signatures. Cutoffs were the same used for all normalization analysis. For example, when 26 of the genes upregulated in the expanded signature (19+7 genes induced in the HNSCC and PCa model) were all upregulated in an individual DTC, that DTC was scored as having 100% of coverage of the dormancy UP genes. The same was applied for downregulated genes. In all cases, differences in means were estimated using a linear regression model and statistical significance was evaluated using t-tests of appropriate model coefficients.

## SUPPLEMENTARY FIGURES AND TABLES


