# Supplementary material for: Characterization of single disseminated prostate cancer cells reveals tumor cell heterogeneity and identifies dormancy associated pathways
Source: Oncotarget. 2014 Sep 16;5(20):9939–51. doi: 10.18632/oncotarget.2480 (PMC4259449; doi:10.18632/oncotarget.2480)
Supplement: Supplementary file 1 [file oncotarget-05-9939-s001.pdf]

Characterization of single disseminated prostate cancer cells reveals tumor cell heterogeneity and identifies dormancy associated pathways

Supplementary Material

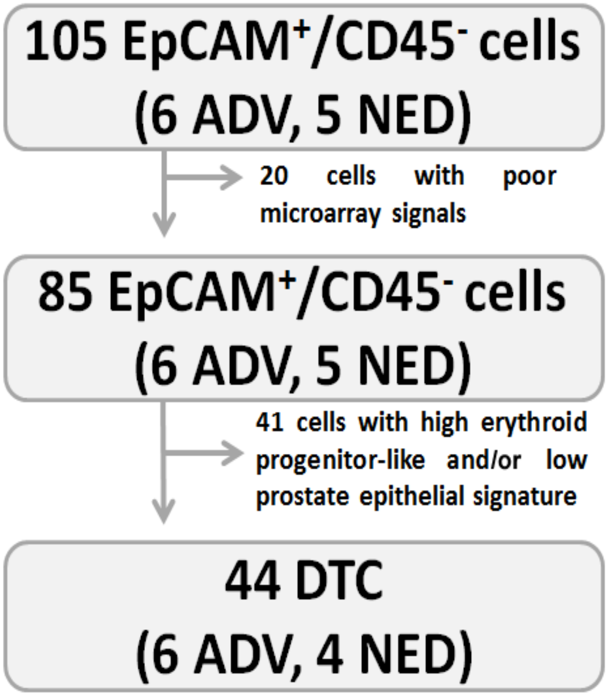

Supplemental Figure 1 : Sample selection in microarray analyses. Microarray profiling of 105 DTC results in 44 DTC that have good microarray quality and high prostate epithelial (low erythroid progenitor-like) signature.

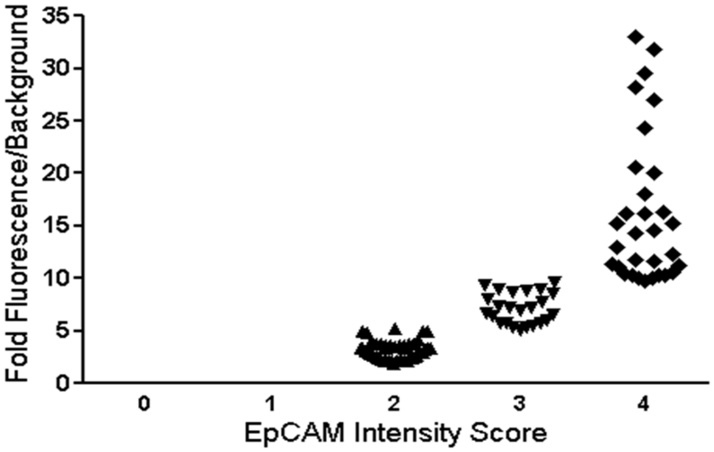

Supplemental Figure 2 : Measuring Epithelial Cell Adhesion Marker (EpCAM) intensity in DTC isolated from patient BM. All cells isolated from the BM are categorized according to EpCAM intensity score based on fluorescence intensity. The graph represents the fluorescence intensity and designated EpCAM Intensity Score using an independent data set of isolated DTC from patient BM (n=76).

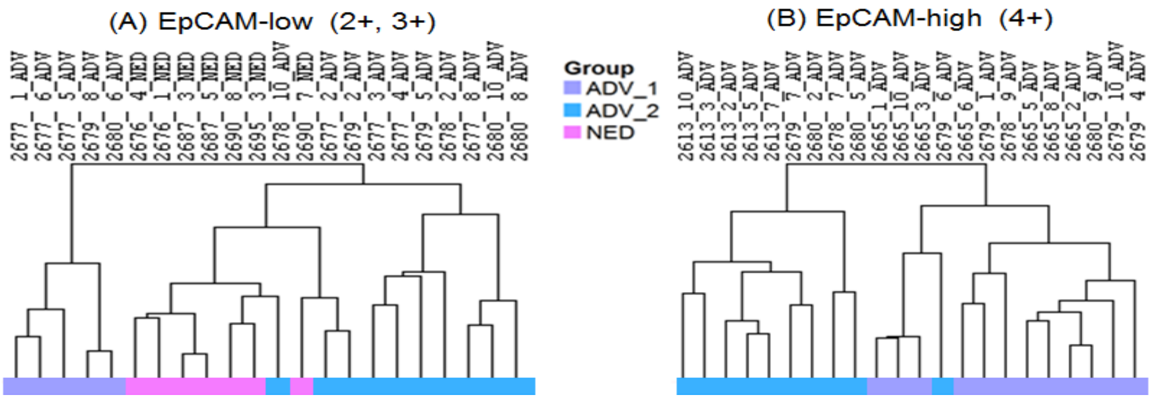

Supplemental Figure 3 : Cluster analysis of the top and bottom 50 most differentiated expressed genes segregates prostate cancer DTC in (A) the EpCAM-low group to 3 categories consistent with NED, ADV_1 and ADV_2, and (B) the EpCAM-high group to 2 categories consistent with ADV_1 and ADV2.

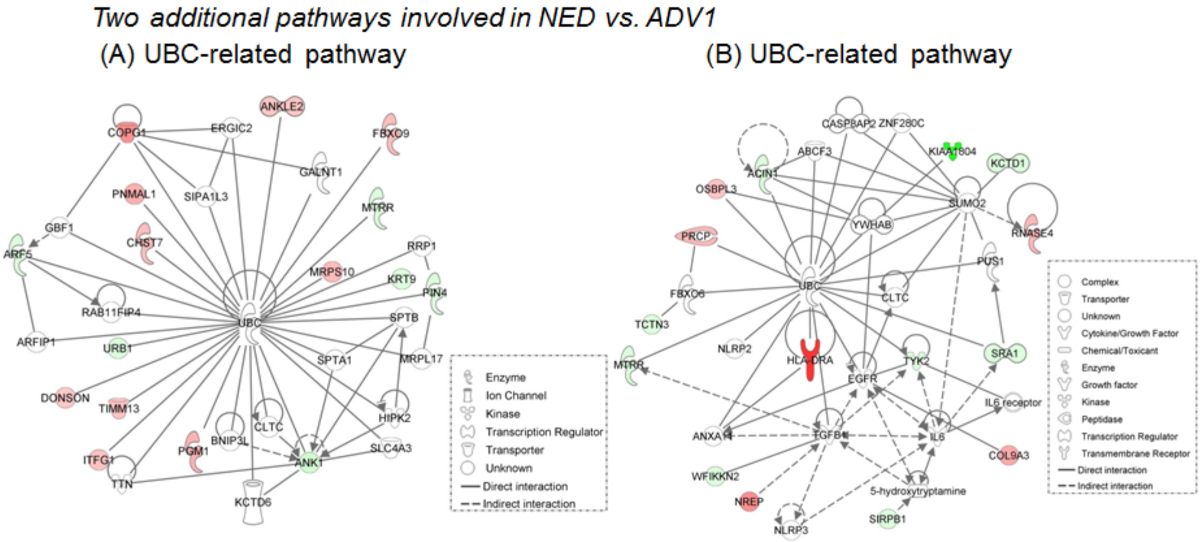

Supplemental Figure 4: Additional pathway analyses displayed differentially expressed genes between no evidence of disease [NED] and a subset of advanced disease [ADV_1] prostate cancer DTC. Ingenuity Pathway Analysis identified two additional pathways associated with ubiquitin C (UBC) altered in addition to the p38 stress response pathway in NED prostate cancer DTC vs. a subset of the advanced prostate cancer DTC [ADV_1]. Red indicates an increase in gene expression, green a decrease. Genes in uncolored nodes were not identified as differentially expressed in our microarray analyzes but were relevant to and therefore incorporated into individual networks based on the IPA database.

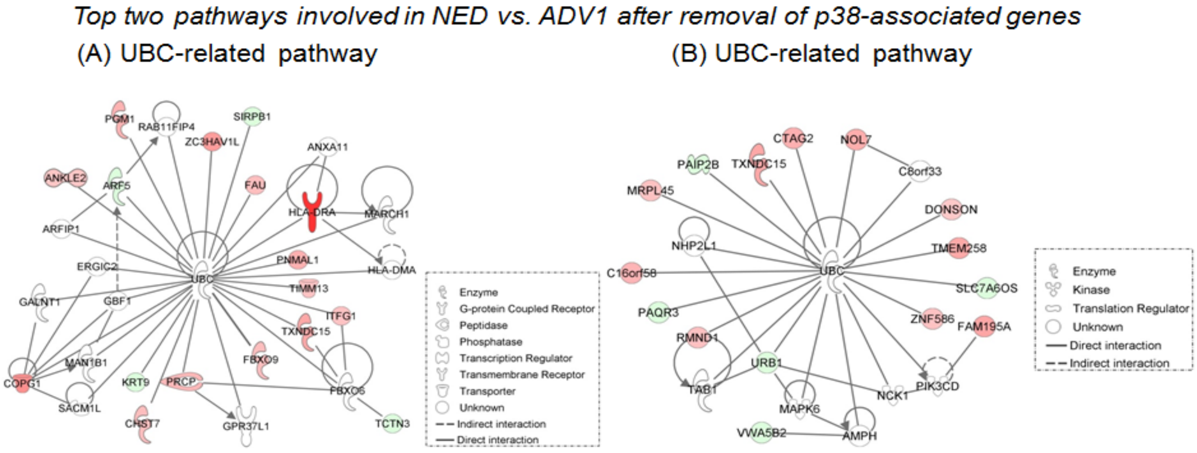

Supplemental Figure 5 : Genes associated ubiquitin C (UBC) pathway are altered between no evidence of disease [NED] and a subset of advanced disease [ADV_1] prostate cancer DTC after removal of p38-related genes. Ingenuity Pathway Analysis identified two top pathways associated with UBC after manual removal of the 21 differentially expressed genes involved in the p38 stress response pathway in NED prostate cancer DTC vs. a subset of the advanced prostate cancer DTC [ADV_1]. Red indicates an increase in gene expression, green a decrease. Genes in uncolored nodes were not identified as differentially expressed in our microarray analyzes but were relevant to and therefore incorporated into individual networks based on the IPA database.

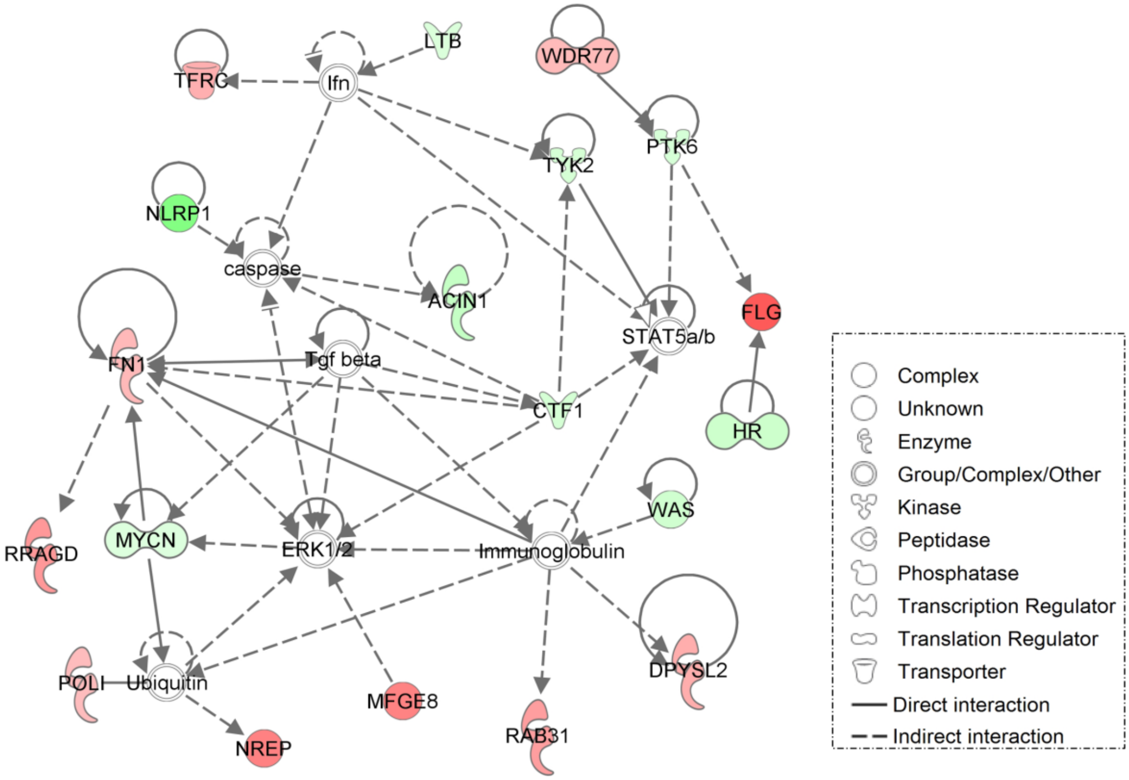

Supplemental Figure 6 : Genes altered between no evidence of disease [NED] and advanced disease [ADV] prostate cancer DTC. Ingenuity Pathway Analysis identified Fibronectin and the ERK pathway was altered in NED prostate cancer DTC vs. all advanced prostate cancer DTC. Red indicates an increase in gene expression, green a decrease. Genes in uncolored nodes were not identified as differentially expressed in our microarray analyzes but were relevant to and therefore incorporated into individual networks based on the IPA database.

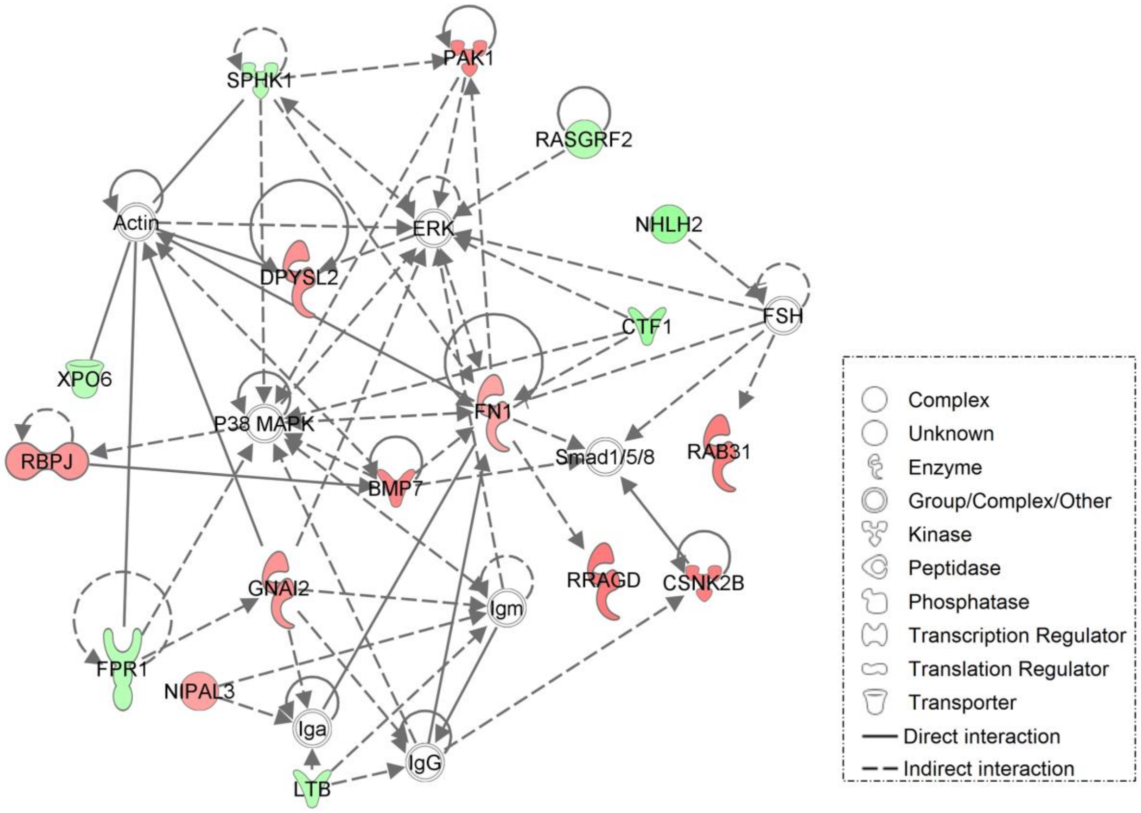

Supplemental Figure 7: Genes associated with Fibronectin, the ERK, and p38 pathways are altered between no evidence of disease [NED] and a subset of advanced disease [ADV_2] prostate cancer DTC. Ingenuity Pathway Analysis identified Fibronectin, the ERK, and p38 stress response pathway as the top biological pathways altered in NED prostate cancer DTC vs. a subset of the advanced prostate cancer DTC [ADV_2]. Red indicates an increase in gene expression, green a decrease. Genes in uncolored nodes were not identified as differentially expressed in our microarray analyzes but were relevant to and therefore incorporated into individual networks based on the IPA database.

Supplemental Table 1: Clinical characteristics of patients with no evidence of disease [NED] and patients with advanced disease [ADV]. PSA; prostate-specific antigen; RP: radical prostatectomy; BM: bone marrow.

NO EVIDENCE OF DISEASE (NED)

	Mean	Min	Max
Age at diagnosis (years)	61.3	58	63
PSA at RP (ng/ml)	4.6	2.6	6.3
Gleason sum		6	7
NED duration (years)	10.8	5.3	18.9
Follow-up (years)	12.6	5.3	19.6

ADVANCED (ADV)

	Mean	Min	Max
Age at diagnosis (years)	64.8	54	78
PSA at diagnosis (ng/ml)	34.6	14	78
Gleason sum		7	9
PSA at BM draw (ng/ml)	604	11	2793
Follow-up (years)	6.8	0.4	18.4

Supplemental Table 2 : Gene listing of the top 50 and bottom 50 genes differentially expressed when comparing DTC from patients with no evidence of disease [NED] and advanced disease groups [ADV_1 and ADV_2]. Gene name is on the left column. Average fold change for the NED, ADV_1, and ADV_2 clusters are given as an average of the log_2 mean centered ratios.

Gene	NED	ADV_1	ADV_2
KIAA1804	-3.2	4.6	-2.8
MTRR	-1.3	1.9	-1.1
NUAK1	-1.3	1.7	-1.0
ANK1	-2.3	3.2	-2.0
ARF5	-0.9	1.3	-0.8
SLC7A6OS	-1.3	2.4	-1.8
PAIP2B	-0.8	1.2	-0.7
SLC25A27	-1.2	1.9	-1.2
FRMD4A	-1.3	1.7	-0.8
ACIN1	-1.5	2.1	-1.2
KRT9	-1.4	1.2	-0.6
WAS	-1.3	0.5	0.0
LOC100130938	-0.9	1.5	-1.0
SRA1	-0.5	0.5	-0.2
CCDC25	-1.2	1.3	-0.7
ZNF177	-0.8	1.2	-0.8
JHDM1D	-1.0	1.2	-0.7
SELK	-0.5	1.1	-0.8
TSPY26P	-1.5	0.8	-0.1
VAMP2	-1.3	0.3	0.2
NLRP1	-2.5	1.4	-0.3
ND6	-1.1	1.1	-0.3
ZFAT	-2.3	1.7	-0.6
TYK2	-1.1	0.6	-0.2
VWA1	-2.2	1.2	-0.2
URB1	-2.2	1.2	-0.3
PAQR3	-0.5	0.7	-0.5
SIRPB1	-1.0	0.5	-0.1
DHDH	-0.8	0.6	-0.2
TCTN3	-0.6	0.5	-0.2
ANKRD16	-0.9	0.7	-0.3
CTSC	-0.7	0.2	0.1
ANP32C	-1.2	1.4	-0.7
TP73	-1.5	0.1	0.4
TPPP2	-0.5	0.4	-0.1
GPR87	-0.5	0.5	-0.3
KRTAP6-3	-1.8	0.6	0.1
C1orf133	-0.3	0.8	-0.6

PHF1	-1.0	0.7	-0.2
SCN9A	-1.6	0.3	0.3
TXNDC2	-0.9	-0.1	0.4
KCTD1	-1.1	0.4	0.1
PIN4	-0.3	0.7	-0.5
ADH1A	-0.4	0.9	-0.6
BRWD1-IT2	-1.0	0.1	0.3
VWA5B2	-1.0	-0.3	0.6
PCDHB9	-2.3	0.3	0.6
HOXB2	-1.2	1.4	-0.8
MSI1	-0.6	0.6	-0.3
WFIKKN2	-1.0	0.4	0.0
COL9A3	0.6	-0.5	0.2
DONSON	0.3	-0.2	0.1
N4BP1	0.8	-0.2	-0.1
FTSJ1	0.3	-0.4	0.3
LBR	0.6	-0.3	-0.1
PGM1	0.7	-0.2	-0.1
CUL9	0.9	-0.4	0.1
SETMAR	0.3	-0.3	0.2
RMND1	0.4	-0.2	0.0
MRPL45	0.5	-0.1	-0.2
ZC3HAV1L	0.6	-0.7	0.3
HLA-DRA	1.9	-0.4	-0.3
ANKLE2	0.3	-0.2	0.1
TIMM13	0.4	-0.2	0.0
PNMAL1	0.6	-0.4	0.1
OSBPL3	0.4	-0.1	-0.1
FAM195A	0.7	-0.5	0.2
ADAT1	1.4	0.0	-0.5
SETD2	0.7	-0.1	-0.1
CREBZF	0.3	-0.3	0.1
ZNF586	0.2	-0.4	0.2
ABI1	0.4	-0.1	-0.1
C16orf58	0.6	-0.3	0.1
NOL7	0.9	-0.1	-0.2
PDIA3	0.2	-0.2	0.1
CDK7	0.3	0.0	-0.1
NUCB2	1.0	-0.4	0.0
C11orf10	0.9	-0.2	-0.2
FAU	0.6	0.0	-0.2
FBXO9	0.7	0.0	-0.2
COX7B2	0.8	-0.2	-0.1
DNAJB9	0.8	-0.2	-0.1
TRNP1	0.5	-0.1	-0.2

PSMB5	0.3	-0.3	0.1
CELF1	0.3	-0.1	0.0
CDC25B	0.3	-0.2	0.0
MLKL	0.6	-0.2	0.0
ITFG1	0.2	-0.4	0.3
TXNDC15	1.0	-0.1	-0.2
TFRC	0.8	0.0	-0.2
PRCP	0.6	-0.2	0.0
CHST7	0.5	-0.2	0.0
MALT1	0.3	-0.2	0.1
MRPS10	0.6	-0.2	0.0
CTAG2	1.0	0.0	-0.3
COPG	1.1	-0.4	0.0
C5orf13	1.4	-0.1	-0.3
NIPAL3	0.7	-0.1	-0.2
RNASE4	0.7	-0.1	-0.2
HERC2P2	0.5	-0.2	0.0
